# Reply to Berry, C. Factors Related to *Bacillus thuringiensis* and Gut Physiology. Comment on “Rajan, V. An Alkaline Foregut Protects Herbivores from Latex in Forage, but Increases Their Susceptibility to Bt Endotoxin. *Life* 2023, *13*, 2195”

**DOI:** 10.3390/life14020207

**Published:** 2024-01-31

**Authors:** Vidya Rajan

**Affiliations:** Department of Medical and Molecular Sciences, University of Delaware, Newark, DE 19716, USA; vidyarajan@hotmail.com

**Keywords:** alkaline guts, transgenic Bt crops, foregut-fermenting herbivores, *Bacillus thuringiensis* δ-endotoxin, susceptibility, pesticidal proteins, insecticidal toxins

## Abstract

The comment titled “Factors related to *Bacillus thuringiensis* and gut physiology” disputes some of the inferences in the paper “An Alkaline Foregut Protects Herbivores from Latex in Forage, but Increases Their Susceptibility to Bt Endotoxin” published in this journal. The key points in the dissent are the following: 1. Bt is generally safe to non-target species. 2. Transgenic Bt crops provide additional ecological benefits due to reductions in conventional pesticide use. 3. Susceptibility to Bt does not indicate alkalinity, nor vice versa. My response is summarized as follows: 1. Bt can form non-specific pores at concentrations of 100 ng/mL in culture, and so is potentially unsafe for animals with gut environments in which Bt persists at or above this level. 2. Initial reductions in insecticide applications have not been sustained and are even increasing in areas planted with transgenic Bt cotton. 3. Acidic guts degrade Bt more efficiently, but I concede that gut alkalinity does not imply susceptibility to Bt due to many factors including resistance in target species, toxin heterogeneity and variable modes of action. However, the susceptibility of foregut-fermenting herbivores with alkaline guts to Bt intoxication cannot be invalidated without further study.

## 1. Introduction

This is a response to a comment titled “Factors related to *Bacillus thuringiensis* and gut physiology” [1] which repudiated some claims made in an article titled “An Alkaline Foregut Protects Herbivores from Latex in Forage, but Increases Their Susceptibility to Bt Endotoxin” [2]. The original article was not intended to litigate the safety of insecticidal toxins or dissect their mechanisms of action, but to put forward a hypothesis for how two completely different herbivore digestive systems could have evolved, and that the difference could relate to a disparity in the amount of latex in their forage. Herbivory pressure in evolutionary terms is largely due to chewing animals such as insect larvae [3], and latex is a protective antifeedant produced by about 20,000 species of flowering plants from more than 40 families [4]. It was in the context of latex’s solubilization in the neutral to alkaline pH found in the anterior digestive chambers of many foregut-fermenters that the proposal that herbivorous insect larvae have alkaline midguts as an evolutionary adaptation to this widespread antifeedant was made. *Bacillus thuringiensis* δ-endotoxins (Bt) are known to target phytophagous insect larvae with alkaline midguts. It was logical to extend the analysis to consider Bt’s effects on other animals with alkaline guts, such as ruminants. Although many trials have shown little effect of Bt across a variety of livestock [5], reports of detrimental outcomes to non-target animals do exist [6,7,8,9,10,11].

## 2. Alkaline Gut pH Protects Herbivores from Latex in Forage

In his comment [1], Berry does not address the major claim in the original paper [2] regarding the selective pressure of latex on the development of alkaline guts in herbivores, except to point to a hypothesis that alkaline guts in Lepidoptera may have evolved to adapt to complex dietary tannins [12]. Several other mechanisms besides alkaline guts protect herbivores from antifeedants, such as proline-rich salivary binding proteins, uncharacterized tannin-binding proteins [13], and microbial symbionts whose populations vary with relative levels of phenolic glycosides and condensed tannins [14]. An alkaline gut also allows Gypsy moth larvae to cope with dietary allelochemicals [15]. Thus, alkaline guts, among other adaptations, may help herbivores to cope with plant antifeedants, including latex.

## 3. Exploring the Claim of *Bacillus thuringiensis* Endotoxin and Its Impact on Non-Targets and Animals with Alkaline Guts

It is the secondary claim of increased susceptibility of animals with alkaline guts to Bt to which Berry registers the following objections [1]:

1. Bt is safe for most invertebrates and vertebrates;
2. Transgenic Bt crops have led to a reduction in the use of conventional pesticides which are associated with detrimental effects on non-target animals;
3. Susceptibility to Bt does not indicate alkalinity, nor vice versa.

These points are addressed below.

### 3.1. The Claim That Bt Insecticidal Proteins Are Not Detrimental to Most Invertebrates and Vertebrates

Berry’s claim [1] that “Bt insecticidal proteins are not detrimental to most invertebrates and vertebrates” is supported by some reports showing safety and specificity [16,17]. On the other hand, morbidity and mortality and histopathological, developmental, microbiome, and food web impacts are reported in non-target animals, and, below, I offer a brief look into these claims. Note that other ingredients in sprayed and dispersed formulations are proprietary, and their effects could not be delineated from that of Bt. Additionally, crystals in dispersals typically require processing by digestive proteases following dissolution, but transgenic crops contain active toxin(s) which do(es) not require further processing for functionality. Finally, the specific types of Bt-crops (e.g., maize, cotton) may vary, and their introduced genes and targets may be different. Please refer to the original publications for details.

#### 3.1.1. The Effect on Morbidity and Mortality

Non-target animals with alkaline guts (cows, sheep, goats, buffaloes, tadpoles [5,6,7,8,9,10]) are reported to be affected by Bt. Lajmanovich et al. (2015) found that the tadpoles of the South American common frog showed lowest observed effect concentrations (LOEC) at 5 µg/mL of the Bt formulation Introban^®^. At 40 µg/mL, 100% mortality was recorded in 48 h [6]. In further investigating the impact of Bti and Btk (*Bacillus thuringiensis* var. *israelensis* and *kurstaki*; specific for Diptera and Lepidoptera, respectively) on the tadpoles of various anurans, Empey et al. (2021) found only a “few studies” and noted the impacts ranging from “few observable effects to acute toxicity” were reported. They conclude: “Nevertheless, under various regimes, these biopesticides may have both lethal and sublethal impacts [.]” [18]. Junges et al. (2017) note that, compared to the highly toxic pesticides temephos and permethrin, Bt showed lower toxicity and higher LOEC on tadpoles but that there was, nonetheless, some toxicity [19].

Boisvert et al. (2000) collated reports of a number of non-target invertebrates, mostly from the same orders as target species, but also of fish, that were affected by Bt [20]. Adding GM Bt176 maize to feed on a German dairy farm was accompanied by a decrease in cow health parameters. There was a proportional increase in cow deaths, from 0% to 40%, when GM Bt176 levels were incrementally raised from 0% to 40% in feed. Deaths reportedly declined with the removal of the Bt maize from feed [7]. Ramdas (2012) [9,10] describes the lived experience of farmers who have grazed ruminants (cattle, goats, sheep and buffaloes) on fields of Bt-cotton, which died or became sick. The specific variety of Bt-cotton was not identified in each case. Vaccinating sheep against PPR and blue tongue reduced mortality, but morbidity remained a problem. Live animals were treated as if they had ingested poisons, and necropsies showed lesions in kidneys, liver and intestines, as well as a 37% increase in the hepatocellular injury marker AST compared to non-transgenic cotton-foraging sheep [9]. The problems described here reveal the gulf between laboratory-based controlled tests which indicate safe levels, and farmers and veterinarians treating obviously sick animals in the uncontrolled real world. Higher mortality had been seen about 10 years ago due to Peste des petits ruminants (PPR) and blue tongue, but, since then, vaccines have become accessible. Sheep are routinely immunized, with consequent lower mortality. This led to the conjecture that morbidity and depressed immune systems made sheep more susceptible to other pathogens (S. Ramdas, personal communication). Stressors such as increased levels of *Pasteurella haemolytica* infections and heavy metals are noted [9,10,21], but other factors such as nutrient quality, temperatures, pregnancy, social dynamics, other pesticides, water access, etc. may exacerbate the impact of the ingestion of Bt-containing feed on field animals, and may not be equivalently reproducible in laboratory-based controlled tests.

For more detail regarding the reports of the intoxication of sheep in published reports [9,10], I contacted the author and veterinarian, Dr. Ramdas, for more information. The scenario she described was as follows: cotton bolls are harvested by mid-January, and the fields of standing plants (which may still carry leftover cotton bolls with seed) are opened up for sheep grazing. Bt levels in the cotton plants were estimated at ~5 µg/g (with established maximum tolerable limits of 5–10 µg/g). It was during this mid-January grazing period following the cotton harvest that shepherds observed morbidity in sheep. Younger leaves emerging after rains, which the sheep prefer, caused higher morbidity than older leaves. Younger sheep were more acutely affected, but chronic exposure appeared to have a cumulative effect [9]. The connection between grazing Bt-cotton and morbidity became so obvious to the shepherds that they now prefer to exhaust other grazing areas first and bring sheep to the Bt-cotton fields in late January rather than mid-January. Such fears were also shared in response to a questionnaire by animal owners in Sudan who saw their sheep and cattle’s health and productivity decline after foraging on Bt-cotton crop residues [8].

In a 2010 study, levels of 1.27 +/− 0.06 µg/mL (~1200 ng/mL) of Bt Cry1Ac protein were found in the postprandial rumen liquor of sheep fed on Bt cotton plants (along with 300 g/day concentrate feed), and 0.25 +/− 0.03 µg/mL (~250 ng/mL) on a 50% Bt cotton diet and 50% other green fodder (+300 g/day concentrate feed) [22]. Oxidative stress was not induced, but rumen histology studies were not carried out. Stumpff et al. (2007) established the levels of Bt required for non-specific pore formation at 100 ng/mL [23], and even lower levels have been reported (described in Section 3.1.2). So, the demonstrated existence in sheep rumens of concentrations of 2.5 times to 12 times the requisite amount for non-specific pore formation is extremely concerning.

It is also pertinent that concerns about experimental designs for safety studies exist. A review of a collection of transgenic Bt and other crops which were tested for an extended period of 216 days did not show changes in the feed consumption or production parameters of a variety of animals. However, toxicological tests recommended for the safety of feed additives or data on organs at slaughter were not obtained due to unfeasibility and experimental difficulties [5]. In a 2020 document titled *Insecticidal Bt crops: EFSA’s risk assessment approach for GM Bt plants fails by design* [24], concerns regarding the congruence of the laboratory assessment of risk compared to real-world application were broached by the organization Risk Assessment of Genetically Engineered Organisms in the EU and Switzerland (RAGES). The European Food Safety Authority (EFSA) rebuts the concerns and stands by its safety findings, but concedes that “EFSA does not consider the use of long-term animal studies with whole food and feed appropriate to explore the safety of GMPs [genetically modified plants] in the absence of specific hypotheses to test” [25]. Rubio-Infante and Moreno-Fierros (2015) conducted an unbiased analysis of Bt’s safety in mammals and concluded that “… they cannot be considered innocuous, as they have some physiological effects that may become pathological [.]”, and called for more trials [26].

#### 3.1.2. Histopathological Impacts

Bt’s specificity to its larval midgut target is related to binding with specific receptors [27,28]. Yet, there are problems with non-specificity or off-target impacts. Hemolytic activity for the Cry15A toxin has been seen due to non-specific pore formation in mouse erythrocytes, and activity increased strongly following trypsin or insect midgut extract treatments [29]. In vitro, Puntheeranurak et al. (2004) show that a 21 kDa fragment produced from a larger 65 kDa toxin at pH 9 makes permanently open cation-selective ion channels in receptor-free planar lipid bilayers at a dose as incredibly low as 4.3 nM (~4.3 × 10^−3^ ng/mL) [30]. pH requirements are also mutable: Grochulski et al. (1995) show that the CryIA(a) toxin can form pores in receptor-free lipid bilayers at pH 6, and their analysis further suggests that arginine:lysine ratios are proportional to the pH requirement for activity. Thus, those toxins with a lower arginine:lysine ratio may have lower pH requirements for pore-formation [31]. Stumpff et al. (2007) show that Bt toxin can “… form large, nonspecific pores under certain conditions, including high toxin concentrations, long incubation times, and relatively low pHs” in membranes lacking receptors altogether at a concentration of 100 ng/mL [23]. Concentrations of 50 and 100 ng/mL of CryIAb toxin did not affect the integrity of primary sheep ruminal epithelial cells held in tissue culture for up to 4 weeks [32], but higher concentrations and longer durations were not tested.

From the in vitro results [23,32], the possibility of even transiently higher membrane-adjacent doses, determined in living sheep fed daily on transgenic Bt cotton ([22], and see Section 3.1.1, above), may cause leakage in tissue layers lacking receptors, which may contribute to a loss in gut integrity. In *L. latrans* tadpoles killed by 48 h exposure to 40 ng/mL Introban^®^, Lajmanovich et al. (2015) found inflammatory infiltration and blood vessel dilation in the intestines [6]. Necrosis in multiple tissues was seen in dead sheep that grazed on Bt-cotton [9,10].

In the “super pest” diamondback moth, *Plutella xylostella*, Sun et al. (2022) propose an “… overarching hypothesis of a versatile mode of action of Bt toxins, which can compensate for the absence of individual receptors, and is consistent with an interplay among diverse midgut receptors in the [Cry1Ac] toxins’ mechanism of action…” [27]. Thus, there is potential for tissue damage with the long-term ingestion and/or high levels of Bt. Further, these analyses do not even consider the pruning effect of Bt on the diversity of symbiotic microbiota in the gut [33] (see Section 3.1.4, below), which might either mitigate or exacerbate toxicity.

#### 3.1.3. The Effect on Developmental Parameters

Gutierrez-Villagomez et al. (2021) measured developmental parameters in *Lithobates sylvaticus* (wood frog) and *Anaxyrus americanus* (American toad) following exposure to VectoBac 200 G (granules) and VectoBac 1200 L (aqueous suspension). They found it did not affect many milestones, e.g., survival, total length and weight, etc., but that both formulations delayed metamorphosis [34]. Glöckner and Séralini (2015) report that cows (number unspecified) whose mothers were fed Bt176 corn showed mammary gland malformation and morbidity [7].

#### 3.1.4. The Effects on Microbiome Composition

Invertebrates have microbiota that are affected by their diet, but this topic is only just beginning to be studied in the context of evolutionary fitness in general [35] and in pest insects [36]. Gutierrez-Villagomez et al. (2021) caution that microbiota changes seen in wood frogs and American toad tadpoles when administered VectoBac 200 G or 1200 L “… could affect susceptibility to parasitic infections…” [34]. Feeding Bt to susceptible western corn rootworms induced dysbiosis to match the less-diverse microbiomes in resistant rootworms [33]. Dysbiosis could potentially occur in non-target animals as well, with unpredictable consequences to gut integrity and health outcomes.

#### 3.1.5. Food Web Impacts

Food web impacts are subtle and enduring. Since target insects are low on the trophic level, they are important in maintaining food web resilience. Belusova et al. (2021) summarize their impact study of Bt application with a figure indicating the direct negative effects on mammals (including humans), pollinators, *caterpillars*, bacteria, *nematodes*, Collembola, fish, tadpoles, Chironomidae, *mosquitoes*, fungi and mycorrhizae (target species italicized). Indirect negative effects accrue in birds, flowers, spiders, parasitoid wasps, ladybugs, lacewings, pirate bugs, fish, dragonfly nymphs, and newts due to a disruption to their food supply [37]. The negative effect on breeding birds was further bolstered by a study by Poulin et al. (2010) showing lower clutches in treated sites [38]. Poulin et al. (2022) examined persistence and found that spore density remained high for 3 years following spraying in wetlands [39]. In their review of the environmental consequences of Bt application, Belousova et al. (2021) conclude their assessment of ecosystem impacts, stating “… the long-reach and long-term consequences of the Bt introduction into nature should be considered…” to explore “… consequences outside the targeted pest species” [37].

### 3.2. The Claim That Transgenic Bt Crops Provide Ecological Benefits Due Reductions in Conventional Pesticide Use

To assess Berry’s claim [1] about the reduced use of (unspecified) broad-spectrum insecticides since the introduction of Bt-engineered crops in the mid-1990′s and the consequent reduction in deleterious effects [on the ecosystem], I turned to the Food and Agricultural Organization’s interactive data website on insecticide use (which did not specify whether broad- or narrow-spectrum). In the US, between 1996 and 2021, insecticide use had indeed fallen from 111,584 tons to 73,772 tons (a 36% decrease). Across the world, however, insecticide use increased in the same period from 2 million tons to 3.5 million tons (a 75% increase) [40]. The banning of certain broad-spectrum pesticides (e.g., DDT) and the development of more potent new ones (e.g., neonicotinoids) makes calculations of tonnage of pesticides deployed vs. lethality complicated.

In *Nature Plants*, Kranthi and Stone (2020) report on a long-term study (~2000–2018) of Bt cotton and pesticide application in India. They find that “Indian cotton farmers today are spending more per hectare on insecticide than they did before *Bt* began to spread [.]” and that “… cotton farmers now face stagnated yields along with ominously rising costs for insecticide [37% more than the pre-Bt high] and increasing costs for seed, fertilizer, irrigation and even herbicide”. They chide those who claim sustainable reductions in pesticide use: “Scientists who study agriculture should know better” [41]. Even in the reference cited in support of reduced insecticide use by Berry [1], between 1998–2011 in the US, genetically engineered (GE) insect-resistant (IR) maize adopters only reduced their insecticide use by 11.2% compared to non-adopters [42]. Therefore, Berry’s claim that deploying Bt-crops has reduced the need for pesticides is upheld in US fields in 2011, but not in those of India in 2018.

### 3.3. The Claim That Susceptibility to Bt Does Not Indicate Alkalinity, nor Vice Versa

Regarding Bt’s preferential activity in alkaline gut animals, the observation that an acidic gut milieu inactivates the toxin was made over 15 years ago. Stumpff et al. (2007) state that “The focus […] has been on monogastric species, where Cry toxins are inactivated by the acidic environment of the stomach. In contrast, [in ruminants] production of active toxin appears theoretically possible…” [23], as well as having been observed in ruminal fluid [22]. Indeed, non-specific pore formation in non-target species may be occurring (see Section 3.1.2, above).

Berry further claims that “the alkaline environment of the gut has no relevance for toxin solubilization” and that “if the role of alkalinity was simply for solubilization […] expression in soluble form [would] make non-targets more sensitive…” [1]. Despite Berry’s assertion, there does appear to be general agreement for alkaline environments for solubilization as a prerequisite for activation by proteases before receptor binding (my emphasis): “Following the ingestion by susceptible insect larvae, inactive protoxins are solubilized in the alkaline and reducing environment of the larval midgut and cleaved into the toxic form by proteases” [32]; “Following ingestion of the inactive protoxin, the crystals are solubilized by the alkaline conditions in the insect midgut and are subsequently proteolytically converted into a toxic core fragment…” [43].

I accept that the claim that alkaline gut animals are more susceptible to Bt must be qualified as “at concentrations with the potential for non-specific pore formation” (Section 3.1.2, above). The concentration at which non-specific pore formation in tissue culture is ~100 ng/mL [23], but more information is required in living animals due to microbiome composition, which may affect Bt toxin activity (see Section 3.1.4, above). Another complication is that some target animals that have developed resistance do not show susceptibility despite an alkaline gut pH. Therefore, I withdraw the claim that “While direct measurement may be difficult in very small animals, there is an indirect way to test the pH of the first chamber of the gut by measuring the organism’s susceptibility to Bacillus thuringiensis crystal δ-endotoxin (Bt)” [2].

## 4. Conclusions

The primary purpose of the original paper [2] was to put forward a hypothesis that alkaline guts may protect foregut-fermenting herbivores from the antifeedant, latex, in forage. This hypothesis has not been contested. The secondary claim that alkaline guts could make animals more susceptible to Bt δ-endotoxins received vigorous repudiation [1]. The specific contentions are the following: 1. Bt is safe to non-target vertebrates and invertebrates; 2. the deployment of Bt has led to lower broad-spectrum conventional pesticide use, and has thereby provided ecological benefits; and 3. that an alkaline gut pH is not indicative of susceptibility to Bt or vice versa.

My response is summarized below:

1. The safety of Bt to non-target animals is *not* established beyond doubt to vertebrates and invertebrates. as claimed by Berry [1]. This is due to the ability of the active toxin to form non-specific pores at concentrations of 100 ng/mL. These high concentrations were detected in the rumen liquor of sheep fed Bt cotton plants as part, or all, of the green forage in their diet.
2. In ecosystems, insecticide applications have only been reduced by 11.2% in transgenic crop fields in the US from ~1998–2011, and have increased in India between ~2000–2018 and worldwide between 1996 and 2021.

In addition, Bt formulations may cause morbidity and mortality in tadpoles. Spores persist for up to 3 years following application, which has unclear impacts on the food web.

3. Although Bt degrades more readily in acidic guts, gut pH cannot be directly correlated to Bt susceptibility as claimed in the original paper [2]. This is due to a plethora of toxins with heterogeneity in their modes of action, and the development of target resistance.

Due to Bt’s potential for non-specific pore formation, the claim of the greater susceptibility of foregut-fermenting animals with alkaline guts cannot be invalidated without further study.
